# Repeated stereotactic radiosurgery for the treatment of relapsed brain metastases: is it time to give up whole-brain radiotherapy?

**DOI:** 10.18632/oncoscience.500

**Published:** 2020-04-24

**Authors:** Luca Nicosia, Vanessa Figlia, Niccolò Giaj-Levra, Giuseppe Minniti, Filippo Alongi

**Affiliations:** ^1^Advanced Radiation Oncology Department, Cancer Care Center, IRCCS Sacro Cuore Don Calabria Hospital, Negrar, Italy; ^2^Department of Medicine, Surgery, and Neuroscience, University of Siena, Siena, Italy; ^3^University of Brescia, Brescia, Italy

**Keywords:** brain metastases, radiosurgery, radiotherapy, linac-based VMAT, whole-brain radiotherapy

Technological progress in radiotherapy (RT) permits the irradiation of brain metastases (BMs) effectively and with limited toxicity. Recently, monoisocentric techniques have been introduced in clinical practice that conjugate traditional stereotactic radiosurgery (SRS) accuracy with the ability to simultaneously irradiate multiple BMs [1]. This has directly led to a significant reduction in treatment time and cost when compared to the conventional radiation approach, which consists of multiple sessions with dedicated plans for each target volume [2,3].

A second advantage of such monoisocentric techniques is maximizing healthy brain radiation dose sparing [3]. As recently reported, this technical ability allows for multiple treatment sessions with minimal toxicity in case of further intracranial progression [4]. The resort to this approach could be strengthened not only by the high efficacy of SRS ablative doses, but also by the lower SRS-related cognitive impairment, as compared to whole-brain RT (WBRT) [5].These emerging data, along with improvement in the early radiological detection of BMs and systemic therapy effectiveness, raise the question of whether WBRT should be still considered a viable treatment option for patients. The most robust data regarding SRS effectiveness are related to cases with ≤10 BMs [6]. Recent prospective and retrospective studies also explored the role of SRS in patients with >10 BMs, which is considered an advanced stage of brain disease [7]. These promising results help to further clarify the reallocation of WBRT. In an attempt to improve WBRT tolerability, a recent phase III trial demonstrated that hippocampal-sparing WBRT plus mementine (an N-methyl-D-aspartate [NMDA] receptor antagonist that blocks excessive pathological stimulation of NMDA receptors, also beneficial for dementia and neuroprotective in preclinical brain irradiation models) resulted in better cognitive function preservation than WBRT plus mementine [8]. Moreover, no difference in oncological outcome was detected.

Another approach that combines the high intracranial disease control provided by WBRT with SRS’ local control is the administration of a dose boost (sequential or simultaneous) to the macroscopic disease [9]. Despite the interest, the results are early and limited to a few retrospective series with little comparison with SRS. Therefore, its use remains experimental.

At our institution, 1,003 BMs in 151 patients were treated with the monoisocentric SRS technique. In limited brain progression cases (usually ≤10 new BMs), a second SRS course was generally proposed, according to patient clinical condition. In selected cases of greater brain disease spreading after an adequate time interval (minimum 6 months), a further course of SRS was also proposed. WBRT was exclusively administered for miliary brain dissemination, or systemic progression no longer suitable for effective systemic treatment [4]. In this case, the best supportive care could also be an acceptable option. The updated results, with a median follow-up of 18 months, revealed that WBRT was recommended in 16 out of 151 patients after a median time of 6 months (range 1-20).

Another important issue is the synergy between new systemic target therapy and SRS. Some of these drugs, including new generation EGFR-tyrosine kinase inhibitors, anaplastic lymphoma kinase (ALK) inhibitors, and immune checkpoint inhibitors, may exert prophylactic action on a healthy brain. This can reduce the occurrence of new metastatic lesions, while SRS can effectively control the macroscopic disease burden. Several tumors, such as HER2 breast cancer, oncogene-addicted NSCLC, and melanoma, might benefit from such an approach.

Despite being mainly limited to retrospective series or post-hoc analyses, current evidence [10,11] suggests that interaction between SRS and new drugs is reasonable, raising the need for large prospective studies.

Meanwhile, the use of WBRT is progressively declining. Future studies will further assess the role of SRS in delaying WBRT. These studies should not only confirm an advantage in neurocognitive function preservation and quality of life, but also demonstrate a survival benefit.
